# cgMSI: pathogen detection within species from nanopore metagenomic sequencing data

**DOI:** 10.1186/s12859-023-05512-9

**Published:** 2023-10-12

**Authors:** Xu Zhu, Lili Zhao, Lihong Huang, Wenxian Yang, Liansheng Wang, Rongshan Yu

**Affiliations:** 1 School of Informatics, Xiamen University, Xiamen, Fujian China; 2 Women and Children’s Hospital, School of Medicine, Xiamen University, Xiamen, Fujian China; 3 Computer Management Center, The First Affiliated Hospital of Xiamen University, Xiamen, Fujian China; 4 Aginome Scientific, Xiamen, Fujian China; 5 National Institute for Data Science in Health and Medicine, Informatics, Xiamen University, Xiamen, Fujian China

**Keywords:** Pathogen detection, Strain identification, Nanopore sequencing, Metagenomic data

## Abstract

**Background:**

Metagenomic sequencing is an unbiased approach that can potentially detect all the known and unidentified strains in pathogen detection. Recently, nanopore sequencing has been emerging as a highly potential tool for rapid pathogen detection due to its fast turnaround time. However, identifying pathogen within species is nontrivial for nanopore sequencing data due to the high sequencing error rate.

**Results:**

We developed the core gene alleles metagenome strain identification (cgMSI) tool, which uses a two-stage maximum a posteriori probability estimation method to detect pathogens at strain level from nanopore metagenomic sequencing data at low computational cost. The cgMSI tool can accurately identify strains and estimate relative abundance at 1× coverage.

**Conclusions:**

We developed cgMSI for nanopore metagenomic pathogen detection within species. cgMSI is available at https://github.com/ZHU-XU-xmu/cgMSI.

**Supplementary Information:**

The online version contains supplementary material available at 10.1186/s12859-023-05512-9.

## Background

Infectious disease is one of the leading causes of death worldwide. In many cases, timely and accurate identification of the exact types of pathogenic microbes is a prerequisite for effective clinical treatment. Traditional clinical pathogen detection relies on culture-based techniques, which are time-consuming and do not meet the need for rapid diagnosis. For this reason, more attention has been paid to the direct detection of pathogens from metagenomic samples recently [1]. Rapid metagenomic testing has been recognized as a promising tool for the diagnosis of unknown infections from body fluids [2]. Recent work showed that it is possible to detect bacterial of lower respiratory infection with high sensitivity on metagenomic samples in 6 h from sample to result based on nanopore sequencing [3].

Genomes of different strains within species are highly similar [4], but subtle differences in genes may manifest as important phenotypic differences relevant to human health. For example, *Escherichia coli strain O57: H7* is pathogenic, whereas *Escherichia coli Nissle* strain is probiotic. Many tools have been developed to identify the precise strain information from metagenomic data, which include three main categories. Assembly-based methods, e.g., BHap [5], STRONG [6] and inStrain [7], can identify new isolates but require a high sequencing depth to ensure the assembly accuracy and are not suitable for low abundance cases. K-mer-based methods, including MetaOthello [8], strainGE [9], Kraken [10] and Kraken2 [11], pre-compute an index of k-mers for each reference genome to classify sequence reads for efficient searching. Mapping-based methods, including MIST [12], snipe [13] and Centrifuge [14], identify specific strains by mapping the reads against an established reference genomic database and evaluate the alignment results.

Next generation sequencing (NGS) typically requires a run time of more than 16 h for most metagenomic studies. In contrast, nanopore sequencing (MinION sequencer by Oxford Nanopore Technologies) can detect microbes within minutes after sequencing starts and has a turnaround time of less than 6 h [15]. Therefore, nanopore sequencing has been considered as a highly potential tool for genomic surveillance of emerging viruses [16–18]. Several tools have been used to analyze nanopore sequencing data, including ORI [19], Centrifuge, MetaMaps [20] and Kraken2. ORI identifies strains from whole genome sequencing (WGS) samples. It only requires a small sequencing depth and achieves good results on samples containing multiple strains. Centrifuge is a rapid and memory-efficient metagenomic reads classifier. It splices different parts of multiple genomes of the same species or genus to form a large genome to alleviate the alignment bias. Unfortunately, it is not able to perform strain level identification. MetaMaps is a reads classifier that identifies strains by mapping the reads to all the reference genomes and analyzing the mapping scores. Kraken2 is a k-mer based method that is not specifically designed for strain level classification. The classification results of Kraken2 rely on the NCBI classification tree. However, most of the genomes do not have independent taxonomy identifiers. Therefore, Kraken2 does not provide satisfactory results at strain level.

A critical step in mapping-based approaches is the alignment of the reads to an established reference genome database. A read may align to multiple reference genomes with a same alignment score due to the high similarity and duplications between strains. In such cases, mapping algorithms may randomly select one as the best result and the rest as secondary alignments [21], which, may lead to incorrect statistical results. This problem is more challenging for nanopore sequencing data due to their high sequencing error rates. To overcome this issue, Bracken [22] counts only the unique reads information of the alignment to improve the accuracy of species-level abundance estimation. Salmon [23] uses the expectation maximization (EM) algorithm to estimate the sequencing level of homologous templates from mappings generated by traditional mappers. KMA [24] is specifically designed for this type of multi-mapping situation. KMA uses k-mer to speed-up mapping and the Needleman–Wunsch algorithm to accurately align extensions from k-mer. Multi-mapping reads are resolved using a novel sorting scheme to ensure an accurate assignment. As metagenomic sequences of different strains from the same species are highly similar, sequencing errors could reduce the accuracy of low abundance pathogen detection from metagenomic samples. To the best of our knowledge, currently there is no tool available to accurately identify strains from nanopore metagenomic data with low sequencing depths, e.g., less than 5× coverage. In addition, mapping nanopore reads is challenging under limited computational resources due to their long read length. For example, MetaMaps requires a long processing time, e.g., over 10 h for 74,000 reads. For these reasons, clinical applications of nanopore sequencing in pathogen detection are still limited.

To overcome these limitations, we developed cgMSI that formulates strain identification as a maximum a posteriori (MAP) estimation problem to take both sequencing errors and genome similarity between different strains into consideration for accurate strain-typing at low abundance. To reduce the computational load, cgMSI adopted a two-stage approach. In the first stage, cgMSI uses the core genome, which is the set of gene alleles shared by all strains of a given species of prokaryotes [25], as a substitute for the whole genomes to quickly identify candidate genomes. The full alignment on the whole genomes is only performed on the selected candidate genome in the second stage for the final strain calling result. We evaluated the performance of cgMSI on synthetic *Klebsiella pneumoniae* datasets and a real sequencing dataset. The results showed that cgMSI can perform accurate strain typing and abundance estimation even at 1× coverage.

## Results and discussion

### Overview of cgMSI and evaluation datasets

**Fig. 1:**
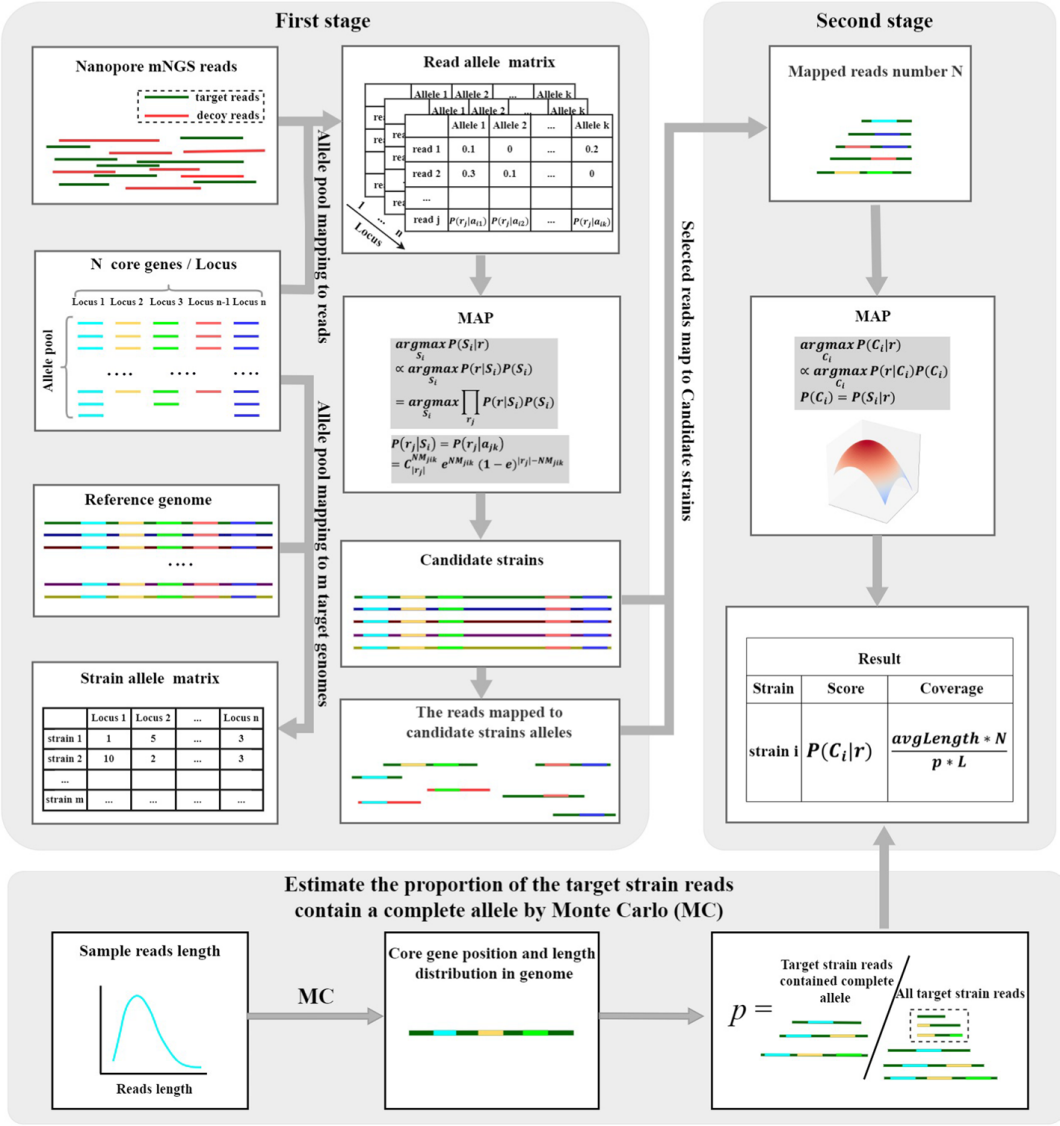
The cgMSI workflow for strain identification on nanopore metagenomic data using two-stage MAP estimation. cgMSI starts by mapping the core gene alleles of the target species to raw reads (using reads as reference) and selects candidate strains using MAP probability estimation. After that, cgMSI maps the aligned reads to the full reference genomes of the candidate strains and identifies the target strain using the second-stage MAP probability estimation. The Monte Carlo method is used to estimate the proportion of the target strain reads containing a complete allele of a core gene, which is further used to estimate the coverage of the target strain

Figure 1 shows the workflow of cgMSI. cgMSI identifies the target strain by a two-stage MAP estimation method. Firstly, the input nanopore raw reads are mapped to a pool of core gene alleles from the target species to calculate the probability that a read originates from different strains per locus. The aligned reads are selected, and candidate strains are identified using the first-stage MAP estimation. Then, cgMSI maps the selected reads against the reference genomes of the candidate strains and obtains the final calling result in the second-stage MAP estimation. We used minimap2 [26] as the default aligner in both stages. Finally, the abundance of the target strain is estimated using the Monte Carlo (MC) sampling method.

**Fig. 2:**
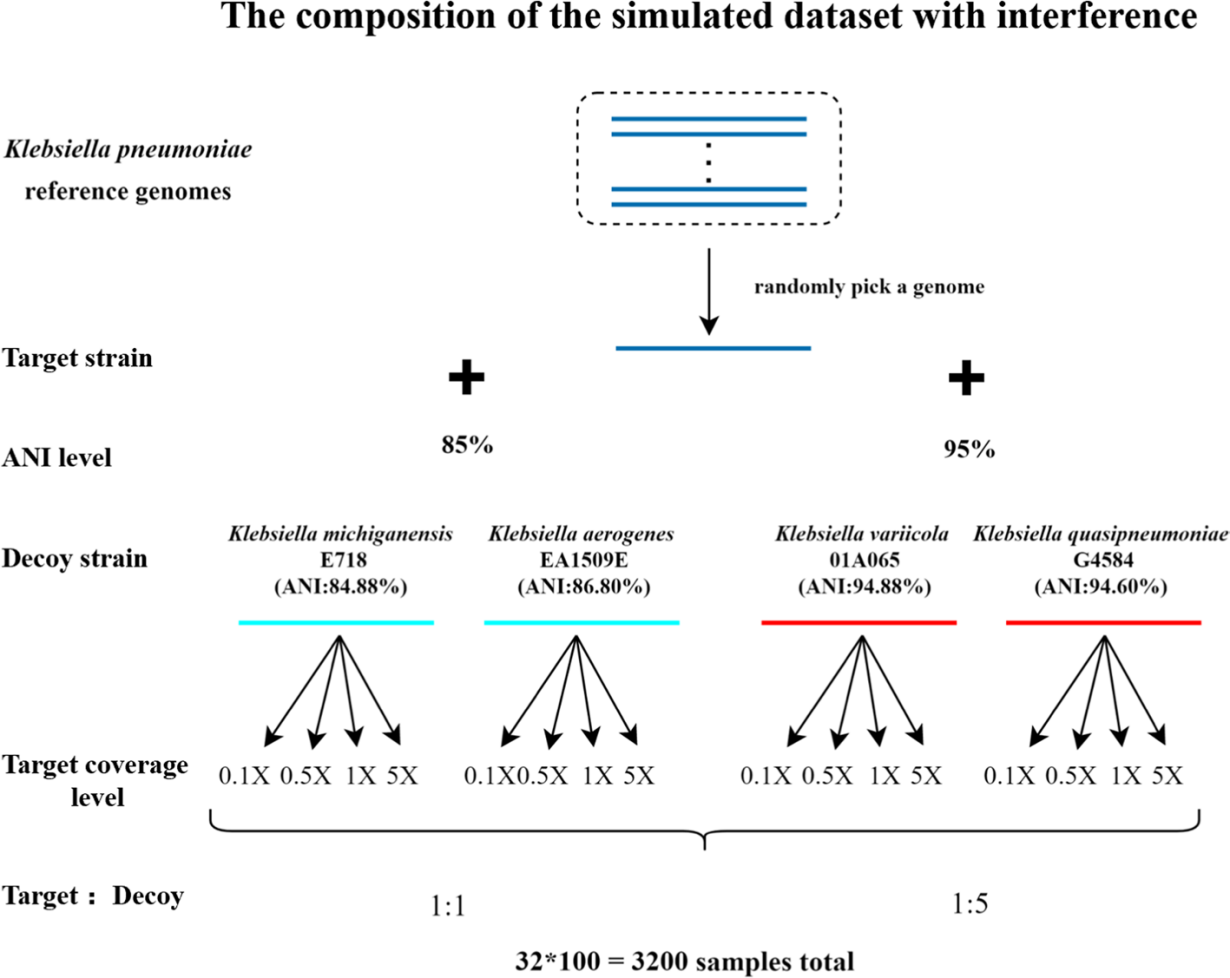
Illustration of the generation of simulated samples with different levels of interference. Each sample contains a random *Klebsiella pneumonia* genome as the target strain and a decoy strain genome. A total of four decoy strains were selected from the *Klebsiella* genus. The pair ANI of the target strain and the background strain was divided into two levels: 85% and 95%. The coverage ratio of the target strain and the decoy strain was divided as 1:1 and 1:5. A total of 3200 samples were generated

We evaluated cgMSI on both simulated and real nanopore metagenomic datasets. We first generated simulated samples with different levels of interference to pathogen detection. We randomly selected 100 strains from the 930 *Klebsiella pneumoniae* strains (available online at the National Center for Biotechnology Information (NCBI) RefSeq [27]) as target strains for synthetic mNGS datasets. For each target strain, simulated reads were generated at different coverage levels (0.1×, 0.5×, 1× and 5×) using NanoSim (version 3.0) [28] with sequencer error profile metagenome_ERR3152366_Log.tar.gz (simulating Flowcell chemistry R9.4) provided by NanoSim and genome mode (-min 1000 -k 6 -b guppy). The simulated reads from target strains were then mixed respectively with simulated reads from four background strains selected from different species under *Klebsiella* at different ratios (1:1 or 1:5) to create the testing samples (Fig. 2).

To simulate mNGS samples of different difficulty levels, we analyzed the genomic similarity of different species under the genus *Klebsiella* and selected four species (KA: *Klebsiella aerogenes*, KM: *Klebsiella michiganensis*, KQ: *Klebsiella quasipneumoniae*, KV: *Klebsiella variicola*) of different Average Nucleotide Identity (ANI) [29] scores ranging from 85 to 95% to the *K. pneumoniae* strains in the database as our background strains. Note that a cut-off ANI score of > 95% between a given pair of genomes is usually considered that they belong to the same species [30]. All ANI values in this paper were obtained using the FastANI tool (version 1.33) [31]. In addition, to evaluate the computational performance of cgMSI, we generated four simulated mNGS samples with different sizes range from 1 to 1000 MB. For each sample, we added one *Klebsiella pneumoniae* strain as the target strain and one *Klebsiella quasipneumoniae* strain as the background strain with the same target strain abundance (20%) for different sample sizes. Here, abundance is defined as the ratio of the number of reads from the target strain to the total number of reads.

To evaluate the performance of cgMSI on complex metagenomic samples, we downloaded 100 nanopore sequencing datasets (NCBI Project ID: PRJNA820119) sequenced from healthy human gut metagenomic samples, and generated simulated samples based on this data. Firstly, we mapped the obtained samples to the high-quality human gut microbiome reference set WIS [32], and removed all reads that mapped to *Klebsiella pneumoniae,* since the human gut was an important reservoir of *Klebsiella pneumoniae*. After filtering, we obtained 100 negative samples. To obtain positive samples, we spiked simulated *Klebsiella pneumoniae* reads into these negative samples. From the available 930 reference genomes of *Klebsiella pneumoniae*, we randomly selected one strain (RefSeq Assembly Accession: GCA_000240185.1) as the target strain. The simulated reads were generated using NanoSim software (version 3.0) with the same settings as described above. To simulate pathogen strains at different sequencing depths, we spiked each negative sample with simulated reads at 0.1×, 1×, and 10×, and obtained a total of 300 simulated positive samples.

For the real dataset, we used the ZymoBIOMICS dataset [33], which contains 8 bacteria and 2 yeasts with equal abundance and was generated on a GridION using the R9.4.1 chemistry for evaluation. Experiments were performed separately on 6 bacterial species with core genes available. For each of the 6 species, we constructed the pool of core gene alleles and reference genome databases, and the corresponding read data were downsampled to simulate coverage levels of 0.1×, 0.5×, 1× and 5× according to the given sequencing depths for each strain. Additional file 2 provides more pathogens information used in simulated dataset.

### Sensitivity of the filtering operation of cgMSI

The first stage of the MAP estimation in cgMSI identifies the candidate strains using core gene alleles. In this filtering operation, we selected the top $K$ strains with the maximum probability as candidate strains. Here, we evaluated the filtering performance with the number of candidate strains $K$ ranging from 5 to 40 on samples with different difficulty levels separately.

**Fig. 3:**
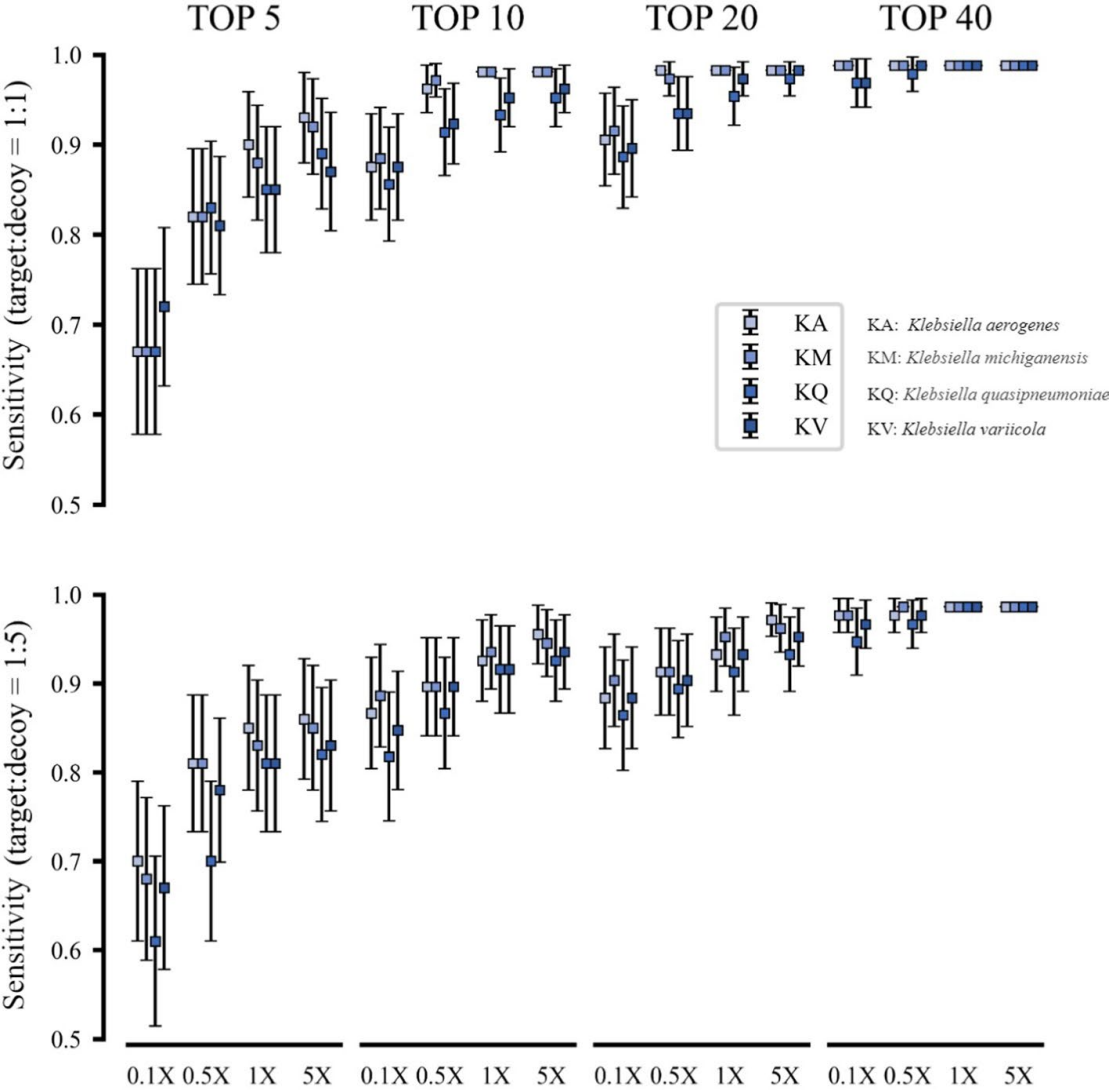
The sensitivity of cgMSI to identify candidate strains using synthetic mNGS datasets. cgMSI identified candidate strains at different coverage ratios ranging from 0.1× to 5×, four different background strains (KA: *Klebsiella aerogenes*, KM: *Klebsiella michiganensis*, KQ: *Klebsiella quasipneumoniae*, KV: *Klebsiella variicola*) and two ratios of target strain to background strain (1:1 and 1:5). Top 5 to Top 40 indicate different numbers of candidate strains identified by cgMSI in the filtration operation. Error bar indicates 95% CI. When the set of candidate strains contains the target strain in a sample, we consider the filtrating operation as correct

Overall, cgMSI correctly identified candidate strains in its first-stage filtering operation in simulated datasets when $K$ was 10 and above (Fig. 3). We noticed that the sensitivity of cgMSI is almost saturated when we identified 40 strains as candidate strains. For each *K* value, the sensitivity was also affected by the coverage, the ratio of the target strain to the background strain (hereafter referred to as the target-to-background ratio), and the difficulty level of the samples as measured by the genomic similarity between the target and the background strains (Fig. 3).

For $K$ greater than or equal to 10, cgMSI correctly identified all candidate strains in KA and KM samples when the target-to-background ratio was 1:1 and the coverage ratio was 1× and above. For more challenging samples (KQ, KV), cgMSI achieved a sensitivity greater than 95% when $K \ge 10$, the target-to-background ratio was 1:1 and the coverage ratio was 1× and above. When the target-to-background ratio was 1:5, cgMSI achieved similar sensitivity performances at 1× and 5× coverage levels, and slightly lower sensitivities at lower coverage levels. However, in the worst case cgMSI still achieved a sensitivity greater than 80% for KQ at 0.1× coverage when identifying 10 or more candidate strains.

### cgMSI identifies strains in synthetic mNGS datasets with interference

We evaluated the strain-level pathogen detection performance using simulated dataset with different levels of interference. For comparison, we used minimap2 + ORI as a benchmark which provided strain-level results in a reasonable timeframe. More specifically, for each sample, we mapped the simulated reads to all *K. pneumoniae* genomes in the reference database by minimap2 to preliminarily filter the reads of the target species, and input these reads to ORI. When ORI outputs multiple predicted strains with corresponding probabilities, we selected the strain with the highest probability as its calling result. We also tried to include MetaMaps in our benchmark test but were not successful due to its slow computational speed at the “map” stage and execution errors at the “classify” stage. In the filtering operation stage, we identified 10 candidate strains which was the default value in cgMSI. Additional file 1: Table S1 provides more information on the software used in the performance comparison experiments.

**Fig. 4:**
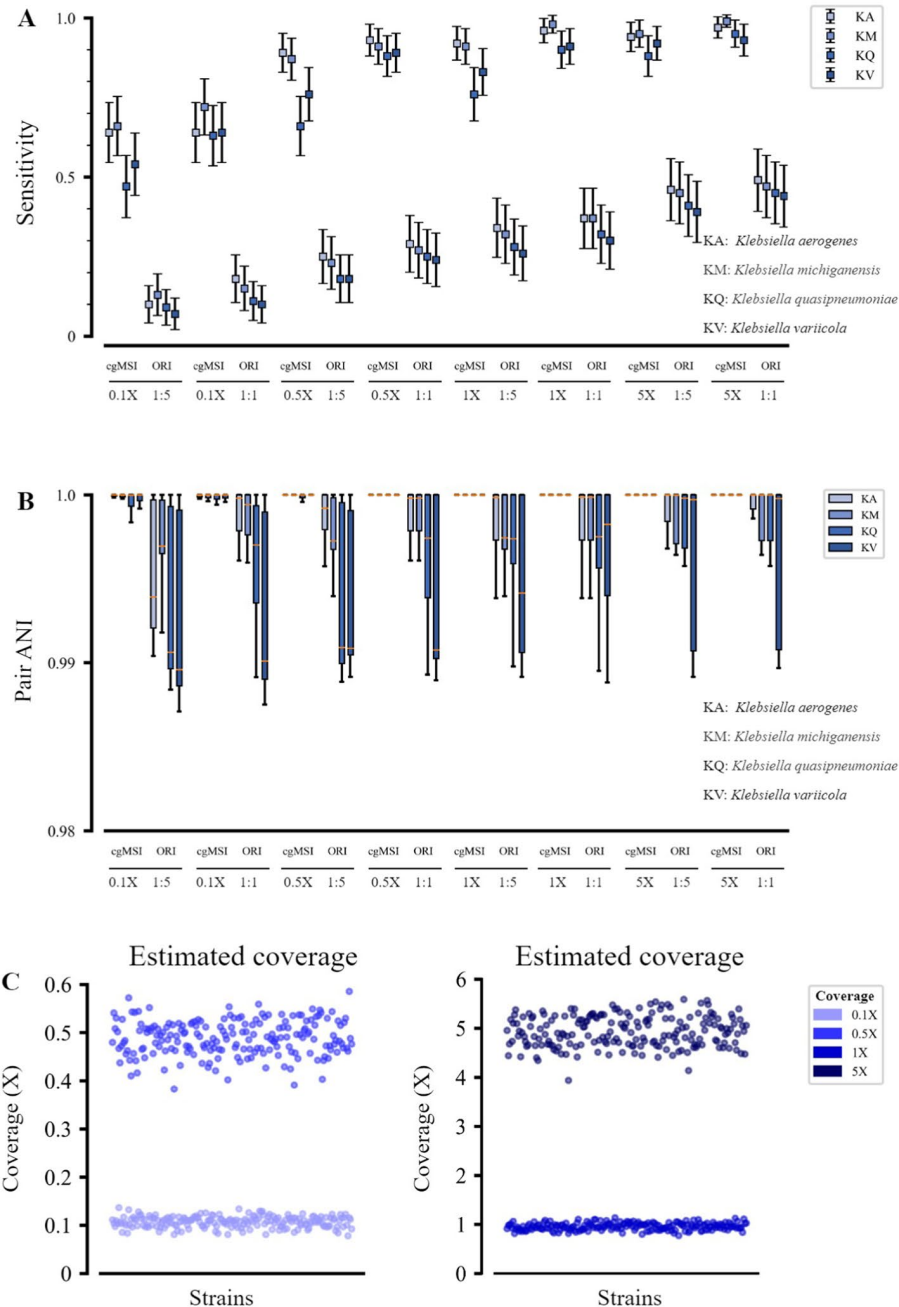
Performance evaluation of pathogen identification using synthetic decoy mNGS datasets. **A** Identification results of cgMSI and ORI from 3200 simulated samples at different coverage ratios ranging from 0.1× to 5×, different background strains and two target-to-background ratios. Error bar indicates 95% CI. **B** The box plots show the pair ANI values of the actual target strain and the predicted strain corresponding to the samples in (**A**). The pair ANI value is used to further assess the accuracy of the prediction. A higher ANI value indicates a more accurate prediction. **C** Coverage estimated by cgMSI at four different coverage levels. Each dot represents a single sample. Colors represent different target strain coverage ratios in the samples

Figure 4 shows the results of cgMSI and ORI for strain-level pathogen detection in synthetic mNGS datasets. Similar to the results for candidate strains identification, it can be seen that the strain typing sensitivity was also affected by the coverage, the target-to-background ratio, and the difficulty levels of the samples (Fig. 4A). Particularly, cgMSI achieved a sensitivity greater than 90% at 1× and 5× coverage when the target-to-background ratio was 1:1. In contrast, the strain typing result showed 40% sensitivity improvement over ORI for all test cases.

For samples whose target strains were not accurately detected, we used the pair ANI value between the actual target strain and the predicted strain to further estimate the typing accuracy. A higher ANI value indicates more accurate strain identification. Note that all samples where target strains were not correctly detected were also typed as strains that were extremely close to the actual strains (Fig. 4B). cgMSI identified all samples with ANI values greater than 0.997. At coverage of 5×, cgMSI achieved an ANI value greater than 0.999. Although the pair ANI value from ORI improved with decreasing task difficulty, there were still some predicted strains far away from the actual target strains (pair ANI < 0.99). The predicted coverage result demonstrated the effectiveness and accuracy of cgMSI for abundance estimation (Fig. 4C).

### cgMSI identifies strains in synthetic human gut metagenomic sequencing data

**Fig. 5:**
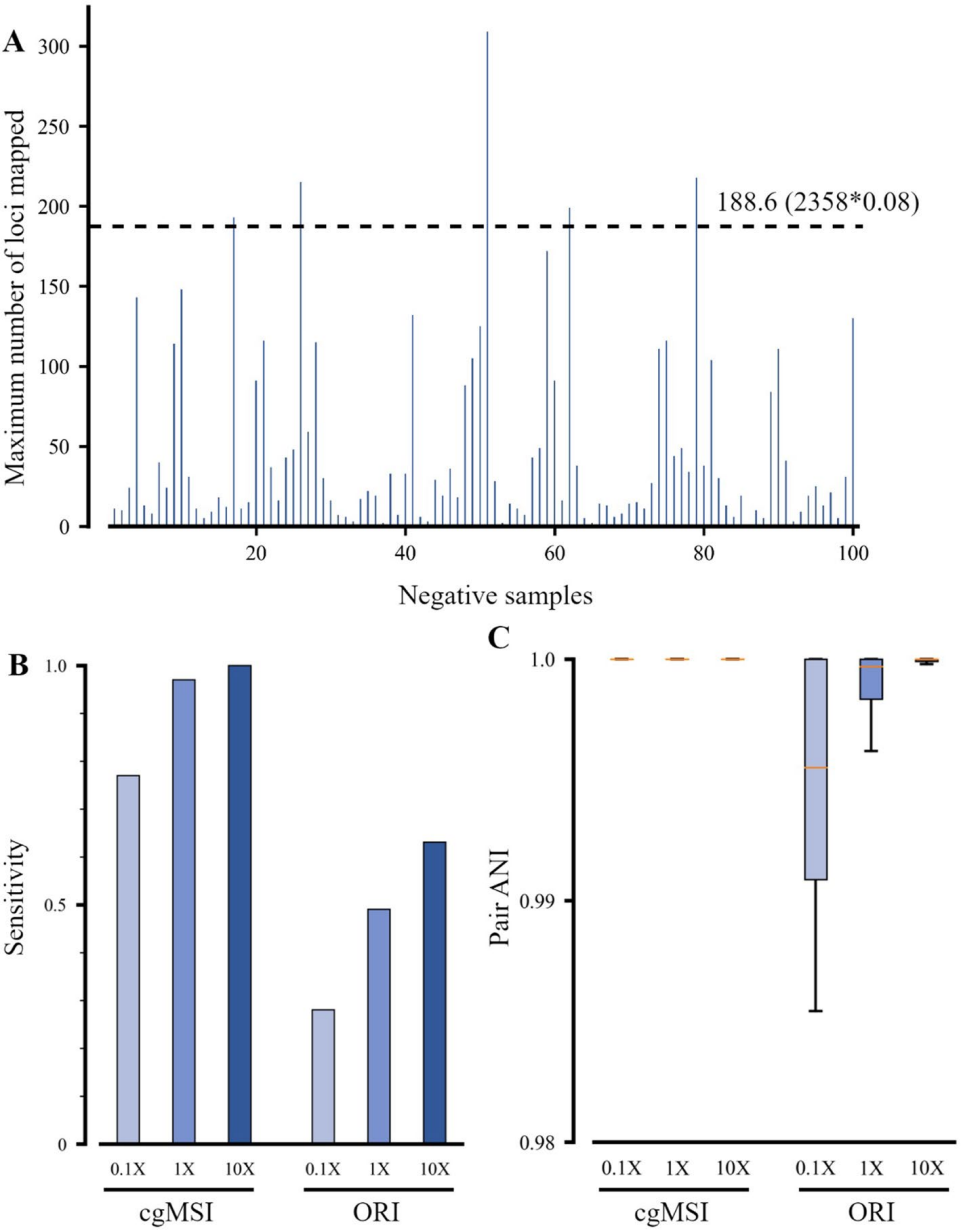
Performance of pathogen identification using synthetic human gut genome datasets. **A** Maximum number of loci mapped in all strains. If the number of loci is less than β times the total core locus number of the target species (2358 for *K. pneumoniae*), the sample is considered to be free of the target pathogen. Here, β uses the default value of 0.08. **B** Identification results of cgMSI and ORI from 300 simulated gut metagenomic positive samples at different coverage ranging from 0.1×, 1× and 10×. **C** The box plots show the pair ANI values of the actual target strain and the predicted strain corresponding to the samples in (**B**)

cgMSI determines whether the target pathogen species is present in the sample by the maximum number of matched core genes among all strains in the first MAP stage, as described in the Methods section. The results showed that cgMSI had a high specificity of 95% for the detection of *Klebsiella pneumoniae* on simulated gut metagenomic samples (Fig. 5A). This indicates that it is more feasible to map sample reads to core gene alleles than to large reference genomes for pathogen identification. The sensitivity of cgMSI was much higher than that of ORI, and both algorithms are strongly influenced by the sequencing coverage of the pathogen strains in the samples (Fig. 5B). On the most challenging samples (coverage of 0.1×), cgMSI had a sensitivity of 77%, which was much higher than the result of ORI (28%). At coverage of 1×, the sensitivity of cgMSI reached 98%, which was close to saturation. For all simulated samples, the pair ANI values of predicted strains and actual strains from cgMSI were greater than 0.999 (Fig. 5C). Among these samples, we randomly selected ten of them to perform Salmonella detection using cgMSI. Additional file 1: Fig. S2 shows that the maximum number of mapped loci in all samples is significantly below the threshold. Furthermore, there is no significant variation in the maximum number of mapped loci for detecting *Salmonella enterica* as the level of spiked-in *Klebsiella pneumoniae* increased.

### cgMSI identifies strains in mock microbial community data

For all species, cgMSI performed well at 0.5×, 1× and 5× coverage ratios. The sensitivity of pathogen strain identification increased with increasing coverage (Table 1). For all samples at coverage 0.5×, 1× and 5×, the average pair ANI of the actual strain and the predicted strain were higher than 0.999. Among the 6 species, cgMSI performed best on *P. aeruginosa* and identified target strains correctly for all samples.

**Table 1:** Performance evaluation using down-sampled ZymoBIOMICS-EVEN dataset

Species	Sensitivity				Average pair ANI of target strain and predicted strain			
	0.1×	0.5×	1×	5×	0.1×	0.5×	1×	5×
*E. coli*	0.7	1.0	1.0	1.0	0.9986	1	1	1
*E. faecalis*	0.6	0.8	1.0	1.0	0.9995	0.9999	1	1
*L. monocytogen*	0.4	0.7	0.8	1.0	0.9976	0.9999	0.9999	1
*P. aeruginosa*	1.0	1.0	1.0	1.0	1	1	1	1
*S. aureus*	0.9	0.9	1.0	1.0	0.9992	0.9992	1	1
*S. enterica*	0.8	1.0	1.0	1.0	0.9984	1	1	1

### cgMSI reduces strain level pathogen detection time

We evaluated the computational performance of cgMSI, ORI, minimap2 and MetaMaps using four different simulated nanopore metagenomic samples of 1 Mb, 10 Mb, 100 Mb and 1000 Mb. For fairness, all tools used the same reference genomes. minimap2 outputs all secondary alignments by control parameter (–N 1000). For MetaMaps, we only counted the CPU time of the “map” stage due to the execution error of the “classify” stage.

**Fig. 6:**
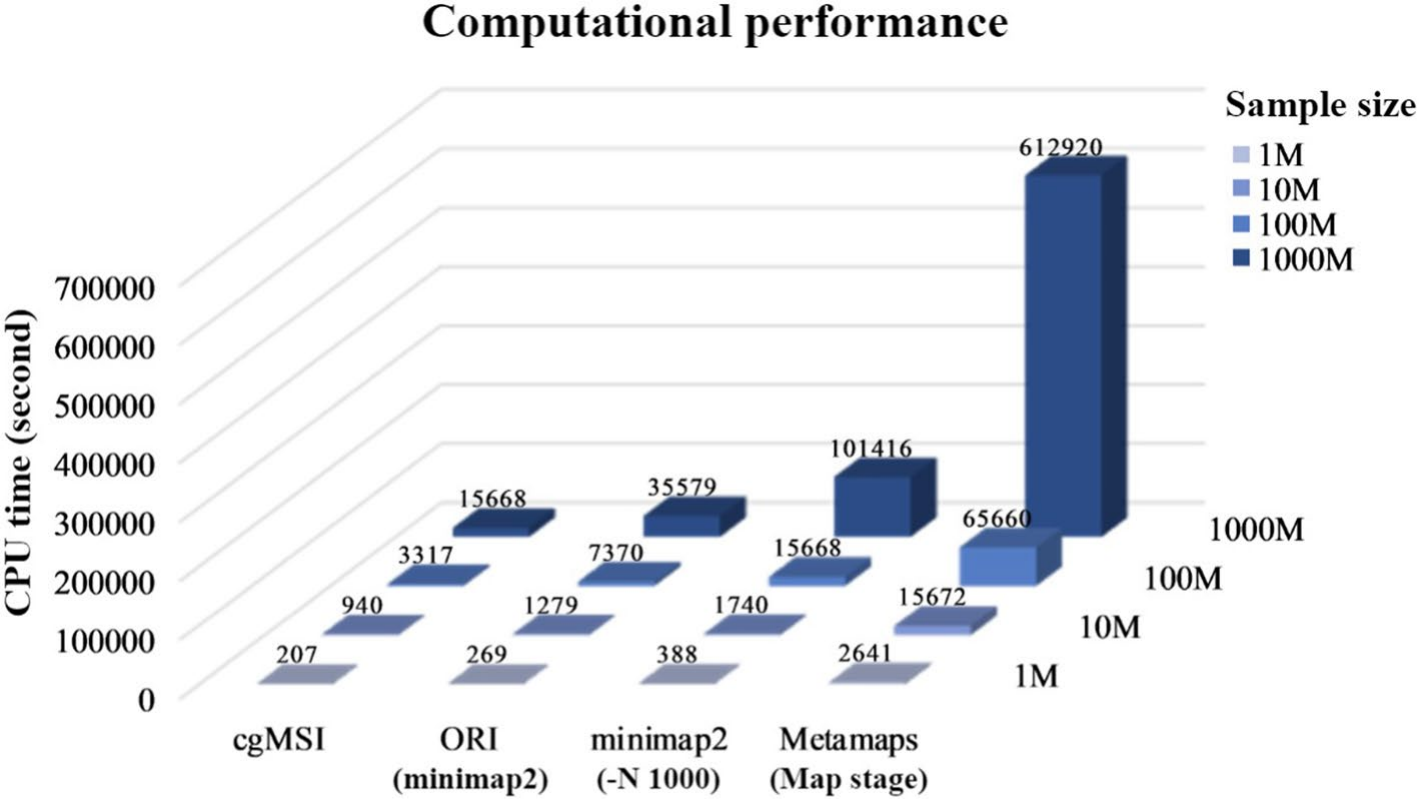
Runtime of different tools at different sample sizes. Runtime performance was evaluated on the simulated metagenomic datasets at different sample sizes with the same abundance of target strain spike-in. Note that the run time of cgMSI consists of three parts, namely, identifying candidate strains, MC sampling, and final result calling. Among them, the process of identifying candidate strains takes the most time. For ORI, we first mapped the samples to reference database using minimap2 to preliminarily filter the reads of the target species, and input these reads to ORI. Minimap2 outputs all secondary alignments by control parameter (–N 1000). For MetaMaps, we only counted the CPU time of the “map” stage due to the execution error of the “classify” stage

Results show that cgMSI outperformed ORI, minimap2 and MetaMaps for all sample sizes range from 1 to 1000 Mb (Fig. 6). MetaMaps took much longer CPU time than the other tools at each size level. When the sample size was 1000 Mb, the CPU time required for cgMSI was 1/2, 1/6 and 1/39 of ORI, minimap2 and MetaMaps, respectively. The fast detection speed mainly comes from the cgMSI strategy of using core genome to identify candidate strains. For the sample sizes of 1 M and 10 M, cgMSI, ORI and minimap2 run with similar time. With the increase of the sample size, the run time increment is much smaller for cgMSI compared to that of ORI, minimap2 and MeteMaps.

We further compared the peak memory used by the four tools (Table 2). cgMSI used the least peak memory for all sample sizes. The peak memory used by minimap2 (–N 1000) increased rapidly with sample size. In contrast, the peak memory used by MetaMaps did not change much file.

**Table 2:** Peak memory consumption for four tools at different samples sizes

Tool	Peak memory at different sample sizes (GB)			
	1 M	10 M	100 M	1000 M
cgMSI	2.14	3.45	9.54	37.14
minimap2 + ORI	0.32	21.23	25.25	1.0
minimap2 (–N 1000)	24.54	25.76	40.43	123.91
MetaMaps	34.37	34.38	36.37	39.87

## Conclusions

We presented cgMSI, an efficient method to identify low abundance strains in nanopore sequenced metagenomic data. cgMSI mitigate the problem of high sequencing error rate of nanopore data by formulate the strain identification as a MAP estimation problem to take full advantage of the information contained in a sample. Furthermore, cgMSI relies on the core genome of a species to filter the candidate genomes and the raw reads containing the core gene before performing full alignment to reduce the computational load of mapping-based strain-typing on nanopore read data. The performance of cgMSI was demonstrated on both synthetic and real datasets. cgMSI software can be used for strain identification withexisting cgMLST scheme, or customized cgMLST scheme generated using software tools such as SeqSphere + (https://www.ridom.de/seqsphere/). Based on its good strain identification performance and fast processing speed, cgMSI can be used to provide valuable reference information in a clinic setting for detecting and managing outbreaks, monitoring pathogen populations, informing treatment decisions, and guiding public health policies, etc. The source code of cgMSI is publicly available and can be downloaded from https://github.com/ZHU-XU-xmu/cgMSI.

## Methods

### Reference database preparation

We obtained the list of alleles for all the core genes of seven common pathogens from cgMLST.org Nomenclature Server (https://www.cgmlst.org/ncs) to create an allele pool of the core genes for each species. The reference genomes for each species were downloaded from the National Center for Biotechnology Information (NCBI) RefSeq [27] (retrieved in December 2021). All genomes are fully sequenced assemblies. More details of the data are given in Table 3.

**Table 3:** The statistical information of the reference core genes and related genomes

Species name	No. of core genes	Ratio of core genes in the genome^a^	No. of genomes^b^
*K. pneumoniae*	2358	0.42	930
*E. coli*	2513	0.48	1858
*E. faecalis*	1972	0.47	68
*L. monocytogenes*	1701	0.52	262
*P. aeruginosa*	3867	0.54	345
*S. aureus*	1861	0.62	671
*S. enterica*	3002	0.59	1021

^a^Plasmid size is not considered when calculating the proportion of core genes in the genome

^b^We downloaded all the complete genomes of a species from NCBI RefSeq as reference for cgMSI

### Select candidate strains by the first-stage MAP estimation

We mapped the allele pool of target species to raw reads of the sample under test using minimap2, and identified a set of reads (r) that can be successfully mapped. Then, the MAP probability of strain $S_{i}$ is calculated as

1 $$\begin{array}{*{20}c} {\mathop {argmax}\limits_{{S_{i} }} P\left( {S_{i} {|}r} \right) \propto \mathop {argmax}\limits_{{S_{i} }} \mathop \prod \limits_{{r_{j} }} P\left( {r_{j} {|}S_{i} } \right)P\left( {S_{i} } \right)} \\ \end{array}$$

For read $j$, we can calculate the probability that it originates from strain $S_{i }$ as

2 $$\begin{array}{*{20}c} {P\left( {r_{j} {|}S_{i} } \right) = \mathop \prod \limits_{k = 1}^{K} P\left( {r_{j} {|}a_{ik} } \right).} \\ \end{array}$$

Here $P\left( {r_{j} {|}a_{ik} } \right)$ is the probability that read *j* originates from allele $a_{ik}$, calculated as

3 $$\begin{array}{*{20}c} { P\left( {r_{j} {|}a_{ik} } \right) = C_{{\left| {r_{j} } \right|}}^{{NM_{jik} }} e^{{NM_{jik} }} \left( {1 - e} \right)^{{\left| {r_{j} } \right| - NM_{jik} }} } \\ \end{array}$$

where $NM_{jik}$ is the editing distance (NM score) of read $j$ to allele $a_{ik}$, $\left| {r_{j} } \right|$ represents the length of read $r_{j}$ and $e$ denotes the sequencing error rate. Here, we selected the top $K$ strains (default 10) with the maximum probability as candidate strains for further identification. During the first-stage MAP, the number of core loci matching the sample is counted for each strain. If the maximum number of core loci matched across all strains is less than β times the total number of core loci of the target species, the sample is considered to be free of the target pathogen. Here, β is a modifiable parameter and its default value is set to 0.08.

### Identify the target strain by the second-stage MAP estimation

Since the length of a nanopore read is typically much longer than the length of a core gene, we mapped the high-quality aligned reads from the previous stage to the complete genomes of the candidate strains using minimap2 to fully utilize the information contained in the reads. The second-stage MAP probability of candidate strains is calculated by

4 $$\begin{array}{*{20}c} {\mathop {argmax}\limits_{{C_{i} }} P\left( {C_{i} {|}r} \right) \propto \mathop {argmax}\limits_{{C_{i} }} \mathop \prod \limits_{{r_{j} }} P\left( {r_{j} {|}C_{i} } \right)P\left( {C_{i} } \right). } \\ \end{array}$$

Here $P\left( {r_{j} {|}C_{i} } \right)$ is the probability of read $j$ mapped to candidate strain $C_{i}$, which can be estimated by [21]

5 $$\begin{array}{*{20}c} {P\left( {r_{j} {|}C_{i} } \right) = \frac{{\exp \left( {MapScore_{ij} } \right)}}{{\mathop \sum \nolimits_{i}^{m} \exp \left( {MapScore_{ij} } \right)}} ,} \\ \end{array}$$

where $MapScore_{ij}$ is the alignment score (AS) given by minimap2 and $m$ is the number of strains that read $r_{j}$ can be mapped to. $P\left( {C_{i} } \right)$ is estimated using the posteriori probability of $C_{i}$ obtained from the previous stage, i.e.,

6 $$\begin{array}{*{20}c} {P\left( {C_{i} } \right) = P\left( {S_{i} {|}r} \right). } \\ \end{array}$$

The strain with the maximum probability is then identified as the target strain.

### Coverage estimated by MC

The coverage of the target strain in the sample is calculated as

7 $$\begin{array}{*{20}c} {Coverage = \frac{avgLength*N}{{p*L}}} \\ \end{array}$$

where $avgLength$ denotes the average length of the sample reads and $L$ denotes the genome length. The number of selected reads that mapped to the candidate strains is denoted as $N$, and $p$ is the probability of a read containing a complete core gene. We estimate $p$ using the Monte Carlo method. In each trial, a simulated read is generated at a random position with a length randomly sampled from length of the raw reads. Let $M$ be the total number of Monte Carlo trials and $C$ be the number of trials where the simulated read covers a complete gene, $p$ is then estimated by

8 $$\begin{array}{*{20}c} {p = \frac{C}{M}.} \\ \end{array}$$

### Data preprocessing

Before the experiments, we performed a quality control on the samples. In this stage, we removed the reads with a length less than 2000 bp or a quality less than 7 using NanoFilt (version 2.6.0). Additional file 1: Fig. S1 shows the results of the quality control, plotted using NanoPlot (version 1.24.0) [34].

### Supplementary Information

**Additional file 1:** contains a supplementary table on software information used in performance evaluation, and supplementary figures on quality control results for simulated samples, and the results of *Salmonella enterica* detection using cgMSI.

**Additional file 2:** provides details of all pathogen reference genomes used in this study.

## Data Availability

Publicly available datasets were analyzed in this study, which can be downloaded from https://github.com/LomanLab/mockcommunity#data-availability. Source code is available at https://github.com/ZHU-XU-xmu/cgMSI.
