# Supplementary material for: cgMSI: pathogen detection within species from nanopore metagenomic sequencing data
Source: BMC Bioinformatics. 2023 Oct 12;24:387. doi: 10.1186/s12859-023-05512-9 (PMC10568937; doi:10.1186/s12859-023-05512-9)
Supplement: Supplementary file 1 — Additional file 1: contains a supplementary table on software information used in performance evaluation, and supplementary figures on quality control results for simulated samples, and the results of Salmonella enterica detection using cgMSI. [file 12859_2023_5512_MOESM1_ESM.docx]

**Additional file 1: Table S1** Software version and options used in performance evaluation.

| **Software** | **Version** | **Options** |
| --- | --- | --- |
| minimap2+ORI | minimap2 version 2.22  ORI version0.0.2 | ‘minimap2 -ax map-ont -t 12 --sam-hit-only’&& ‘howdesbt makebfQ --k=15’ && ‘howdesbt queryQ –sort --threshold=0.5’. Other parameters in ORI used default set. |
| MetaMaps | version 0.1 | ‘metamaps mapDirectly -t 12 --all --maxmemory 60’ |
| minimap2 (-N 1000) | version 2.22 | ‘minimap2 -t 12 -N 1000 -ax map-ont –-sam-hit-only’ |


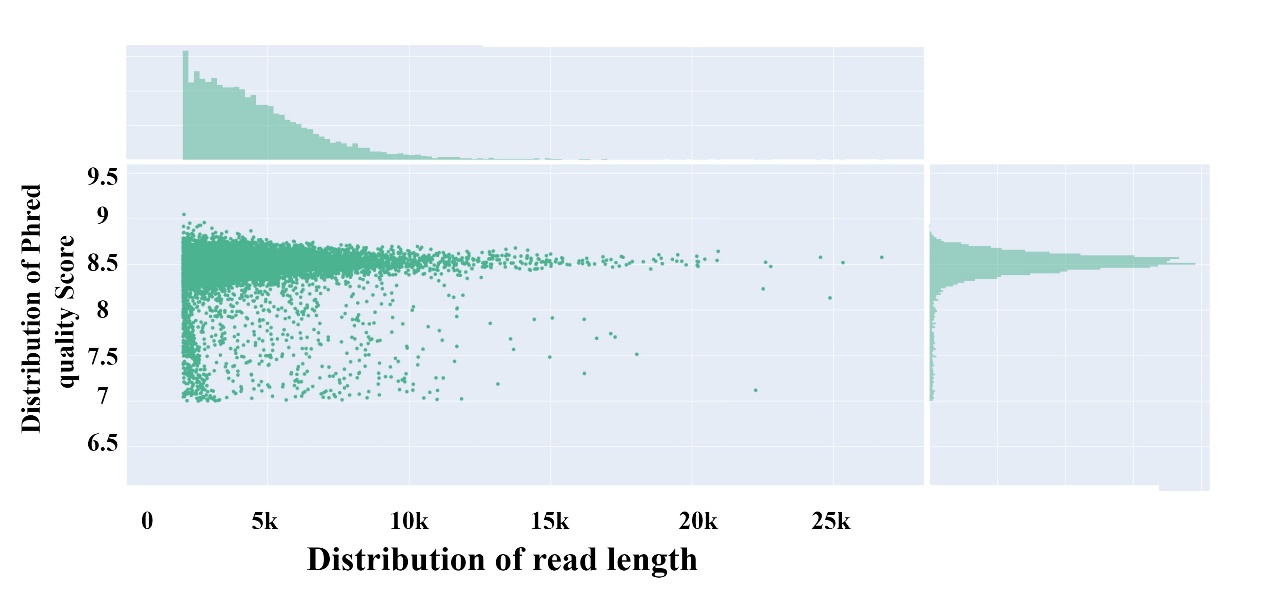


(a)


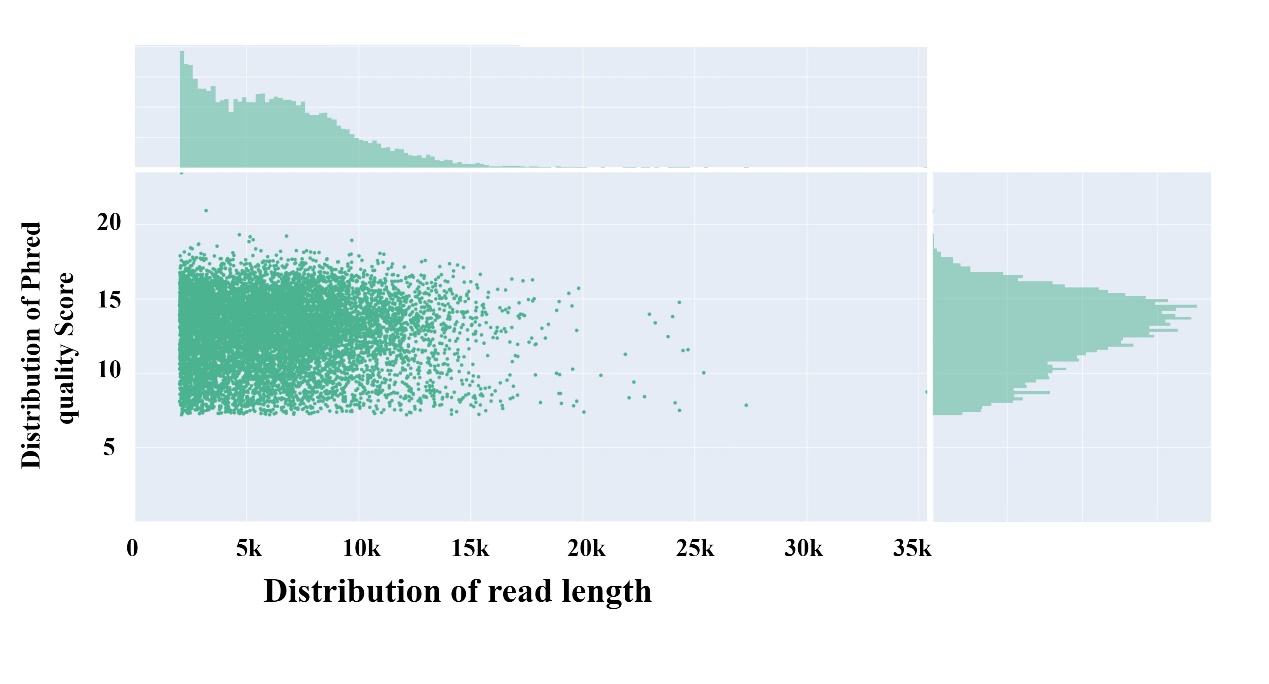


(b)


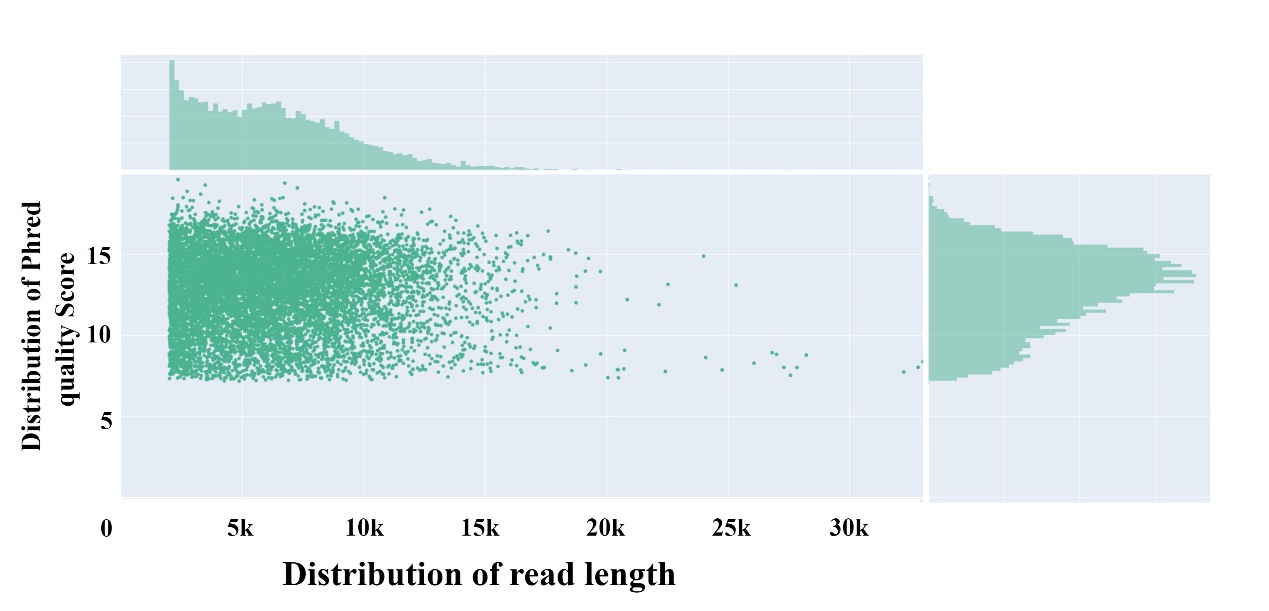


(c)

**Additional file 1: Fig. S1** Quality control result of (a) simulated samples with interference, (b) simulated human gut metagenomic samples, and (c) the ZymoBIOMICS-EVEN dataset. The quality control criterion was to remove reads with length less than 2000 bp or quality less than 7.


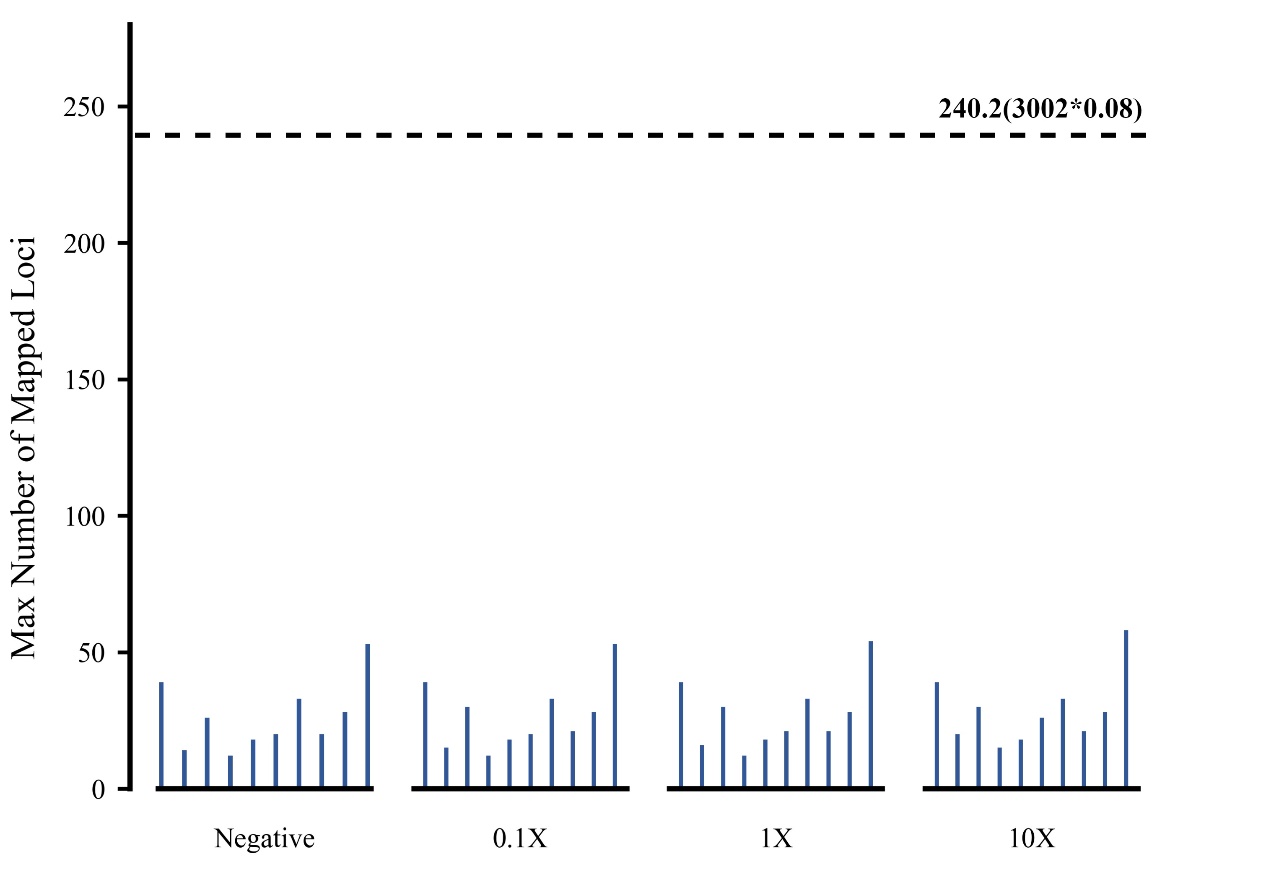


**Additional file 1: Fig. S2** Result of detecting *Salmonella enterica* by cgMSI using human gut genome datasets with different coverage of *Klebsiella pneumoniae* spike-in. The x-axis represents negative samples and samples with 0.1X, 1X and 10X *K.pneumoniae* spiked-in respectively. The y-axis represents the maximum number of mapped to *Salmonella enterica*. If the number of loci is less than β times the total core locus number of the target species (3002 for *Salmonella enterica*), the sample is considered to be free of the target pathogen. Here, β uses the default value of 0.08.
